# Objective Analysis of Movement in Subjects with ADHD. Multidisciplinary Control Tool for Students in the Classroom

**DOI:** 10.3390/ijerph17155620

**Published:** 2020-08-04

**Authors:** Mireia Sempere-Tortosa, Francisco Fernández-Carrasco, Francisco Mora-Lizán, Carlos Rizo-Maestre

**Affiliations:** 1 Department of Computer Science and Artificial Intelligence, University of Alicante, 03690 Alicante, Spain; mora@ua.es; 2 Development Psychology and Teaching Department, University of Alicante, 03690 Alicante, Spain; francisco.fernandez@ua.es; 3 Department of Architectural Constructions, University of Alicante, 03690 Alicante, Spain; carlosrm@ua.es

**Keywords:** assessment, diagnosis, ADHD, motion capture, hyperactivity, building

## Abstract

The term Attention Deficit Hyperactivity Disorder (ADHD) has a long history of problems behind it. The origin of all these problems lies in the lack of agreement in the assessment procedures and evaluation instruments. The diagnosis is clinical and is determined by the observation and information provided by parents and teachers. So, this is highly subjective and leads to disparate results. Therefore, on the one hand the inaccuracy of the diagnosis of ADHD, which has been based on subjective criteria, together with the fact that hyperactivity is one of the main symptoms of this disorder, implies that several studies (with limitations) have been carried out to record objective measures of movement in subjects in at least the last ten years. In order to solve some of this derived problems and limitations of previous studies, a computer program has been developed to objectively record the amount of movement of subjects. The main objective of this study is threefold: first to register the amount of movement of both experimental group and control group, then to compare them with the movement registered by observers and finally to determine the validity of the software developed as a tool to support the diagnosis of ADHD. Results show that there are significant differences in the amount of objective movement between a clinical group of subjects with ADHD and a control group, obtaining a higher average of movement the experimental group. In addition, results also demonstrate that the developed software is a valid tool for the evaluation of movement that solves the limitations of previous studies. The proposed tool is developed from different aspects to give it a multidisciplinary character.

## 1. Introduction

The term Attention Deficit Hyperactivity Disorder (ADHD) is used by the American Psychiatric Association in the Diagnostic and Statistical Manual of Mental Disorders [1] to describe a persistent pattern of inattention and/or hyperactivity-impulsivity that is inconsistent with developmental level, and that impacts negatively and directly on social and academic/occupational activities. We are thus situated in the last interpretation of an old problem that, although highly topical, has a long history of queries behind it.

The problems related to ADHD not only come from outside the scientific community, but have also spread within itself: multiple doubts in reference to etiology, prevalence, evaluation instruments and procedures, etc. The key to all these problems lies in the lack of agreement when considering diagnosis. Thus, in the absence of any biochemical, structural or genetic condition that unequivocally determines the existence of ADHD, the diagnosis is clinical, that is, based on the professional expertise of the doctor, and it is determined by the observation and information provided by parents and teachers [2]. This is highly subjective and leads to disparate results, largely due to the lack of agreement in the assessment procedures and evaluation instruments [3]. In addition, as it has been stated by the Spanish Association of Neuropsychiatry, “the main problem is that the clinical criteria used to diagnose this disorder are too vague. The diagnostic manual used, DSM-V, includes very broad and subjective criteria. How can be determine if a child is more or less prone to a higher degree of movement?” [4].

Consequently, the inaccuracy of the diagnosis of ADHD, based on subjective criteria [5], together with the fact that hyperactivity is one of the main symptoms of this disorder [6,7,8], means that, for more than a decade, several studies have been carried out to record objective measures of movement in the subjects. However, these studies have a number of limitations. First of all, the use of accelerometers (actigraphy and inertial measurement units), that is, that some device has to be placed in the body of the subjects, limiting their ecological validity and, secondly, the use of infrared devices that only analyse some parts of the body [9,10,11,12,13].

The present paper tries to fill the aforementioned shortcomings by using Microsoft Kinect V.2. This device is able to completely track and record 25 joints of six human bodies through an infrared system, without placing any type of sensor in the body of the subjects [14,15,16,17,18,19]. The commercialized version of this device allows developers to make their own programs by using gestures and body movements in a wide variety of applications (Ding and Chang [20]) besides, some emerging researches on the use of this device (that enable us to count limb movements in subjects with ADHD) conclude that it is a good device to measure movements [21]. Furthermore, since this is a device of small dimensions, it can be introduced into the natural environment of the subjects.

For all the above, and trying to contribute to broaden the knowledge about the behaviour of subjects with ADHD, a computer program has been developed to objectively record the amount of movement of the subjects. Subsequently, study techniques workshops have been designed as an incentive used for attendance, both for subjects with a firm diagnosis of ADHD and for control subjects.

The main objective of this study is to introduce a multidisciplinary tool developed for objective analysis of movement in subjects with ADHD. A computer program was developed to record the amount of movement of subjects. The validity of this software as a tool to support the diagnosis of ADHD has been carried out by comparing the amount of movement of the group diagnosed with ADHD and the control group, and it has also been compared with the movement registered by observers.

## 2. Materials and Methods

### 2.1. Participants

This research involved 65 children, aged between 7 and 12 years old. The children were classified into two groups: experimental group and control group. The experimental group consisted of 32 children with a firm diagnosis of ADHD: 24 boys and 8 girls. In total, 24 of the 32 children had a diagnosis of ADHD with a combined presentation and 8 with a predominantly inattentive presentation, according to the DSM-V criteria. The age range of the participants of the experimental group was 7 to 12 years old (M = 9.75 and SD = 1.50).

The control group consisted of 33 children from a standardized sample: 17 boys and 16 girls. The age range of the participants in the control group was 8 to 12 years old (M = 9.69 and SD = 1.50).

### 2.2. Procedure

#### 2.2.1. Sample Recruitment

The project was presented to the Ethical Committee for Clinical Research (CEIC) of the General University Hospital of Alicante (HGUA), obtaining its approval. From that moment onwards, informed consent to participate in the study was given to the parents of children diagnosed with ADHD by the Children’s Mental Health Unit (USMI).

Once the procedure for the recruitment of the sample of the experimental group was started, a primary education school was contacted for the recruitment of the sample for the control group. The same number of boys and girls were randomly recruited as the experimental group.

#### 2.2.2. Study Techniques Workshops

The first step for the organization of the study techniques workshops was to look for an only space, since the attendees (the sample) came from different educational centres. We searched for a classroom at the University of Alicante (UA) with writing pad chairs, because it would greatly facilitate the recording with the Kinect device.

The different sessions of the workshops were organized by grouping children on age basis and ensuring that there were children from both the control group and the experimental group in the different workshops. Only between 4 and 6 people attended to each of the workshops, due to the limitations of the Kinect device (it can only detect and track 6 bodies).

Families of children sent by the USMI that were on some type of medication, were invited to attend a new workshop to “review and expand” study techniques. Second to this workshop, they had to attend without having taken any medication. The families showed interest in collaborating, since all of them agreed, so that we could have all the subjects of the experimental group that has been prescribed some medication within two measurements of movements: with and without medication.

#### 2.2.3. Classroom Layout

**Figure 1:**
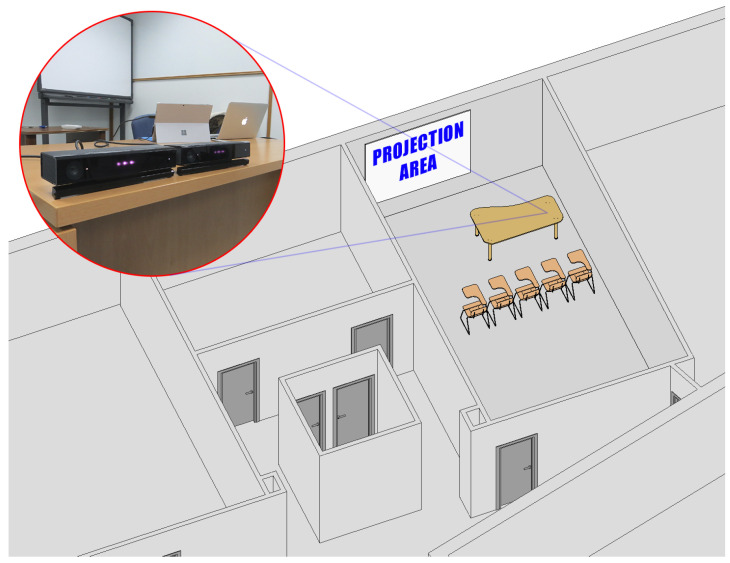
Arrangement of Kinect devices in the classroom. Space previously analyzed with BIM (Building Information Modelling) tools.

Two Kinect devices were used in each workshop in order to ensure the capture of movement of the subjects and prevent any type of technological incident Figure 1.

**Figure 2:**
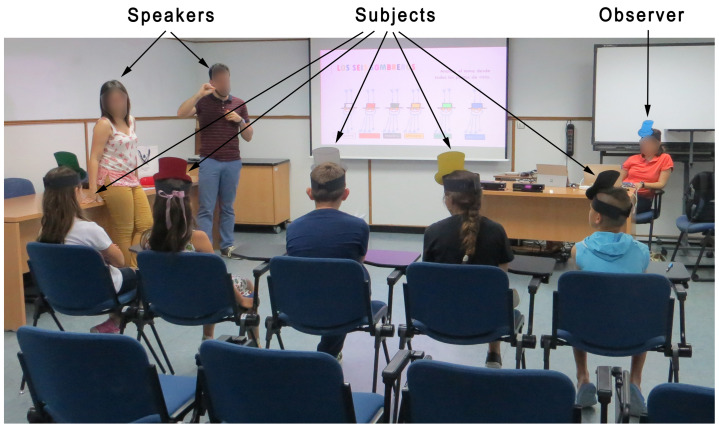
Classroom layout in the study techniques workshops.

The classroom was arranged in such a way that the speakers never stood in front of the Kinect devices (Figure 2). Thus, the speakers would cross behind the chairs when it was necessary to hand out any type of material to the assistants or to answer any question.

An observer attended each workshop together with the subjects and the speakers. Her role was to take notes on the subjects’ behaviour so that, in addition to having the objective movements of the subjects, the appreciations of an independent observer would be registered. Two of the behaviours registered by the observer, along the lines of the diagnostic criteria of the DSM-V and the World Health Organization [22], were the times that the subjects left seat and squirmed in seat. These behaviours were registered in a template that had previously been arranged.

#### 2.2.4. Computer Application ADHD Movements

**Figure 3:**
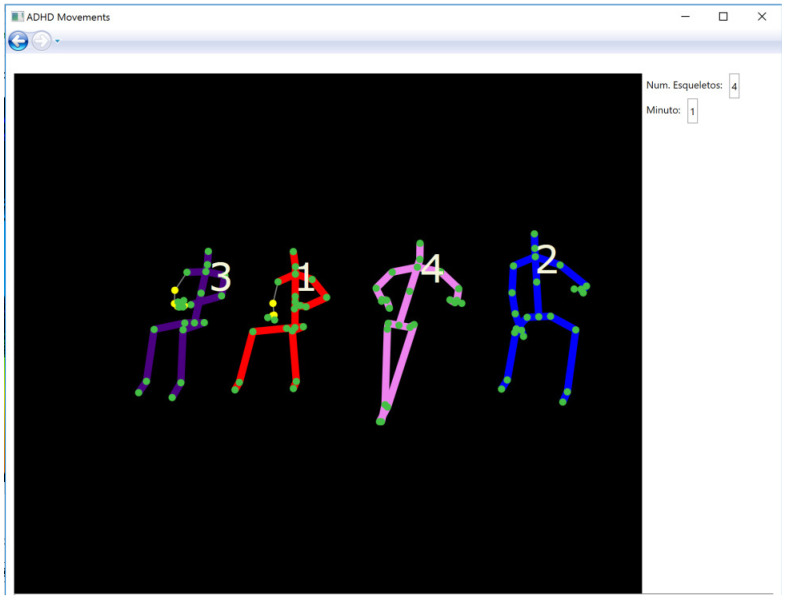
Screenshot of the application *ADHD movements*.

The computer application *ADHD Movements* was developed for the analysis of the movement of several subjects using Kinect. The version of Kinect for Windows allows developers to make their own programs using gestures and body movements in a wide variety of applications, for this reason it is considered an innovative interaction interface between people and computers, in addition to its simplicity in the acquisition of data [20]. This application was developed with Microsoft Visual Studio and using the Kinect for Windows Software Development Kit (SDK) 2.0 that enables the creation of applications that support gesture recognition. For this, one of the main characteristics of this SDK is the detection of the human body that it is represented as a skeleton consisting of segments (such as forearm, hand or foot), which are connected to each other by joints (such as elbow, wrist or ankle). *ADHD Movements* detects the number of visible skeletons and, to allow the observer to know if the detection is correct, it shows each skeleton and each joint on the screen Figure 3.

The application numbers each skeleton (from 1 to 6) in order of detection and saves its location in the environment. In this way, if for any reason, a skeleton stops being detected (for example, a subject stands up and leaves the detection zone), when it returns to its location, the application assigns it the same number.

**Figure 4:**
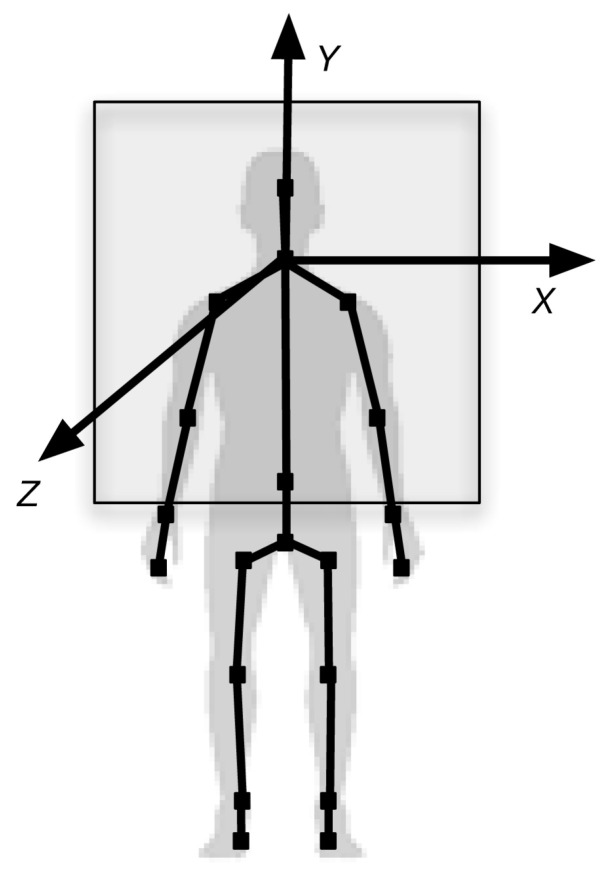
Coordinate system with respect to the skeleton detected.

Data obtained by the Kinect sensor includes information of depth measures and, therefore, of the coordinates (*x*, *y*, *z*) of the objects present in the scene. In the case of a human body, it obtains the coordinates of each of the 25 joints that it detects for each skeleton. Each joint is identified by its name and position and it is measured with three coordinates (*x*, *y*, *z*) where (*x*, *y*) define the position and (*z*) represents the distance to the sensor, according to the coordinate space shown in Figure 4.

Using this information, the developed application estimates the distance covered by each joint of the skeleton in a three-dimensional space during each minute of the session for each of the subjects. Thus, for each joint, the distance between the different 3D points of the trajectory of the joint in the movement was calculated for each minute of the session.

**Figure 5:**
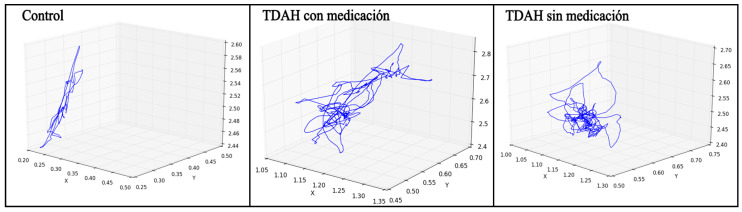
Trajectory of head joint for a control subject (**left**), an ADHD subject with medication (**centre**) and the same ADHD subject without medication (**right**).

As an example, Figure 5 shows the trajectory of the head joint for one minute of a subject with a firm diagnosis of ADHD (centre), the same subject without medication (right) and a subject in the control group (left).

The application obtains, for each subject, a table that shows for each joint the amount distance that it moves in each minute of the session. Sources of this computer program can be downloaded from the software repository of the research group located at https://web.ua.es/en/iamswarm/repository/sw-repository.html.

### 2.3. Analysis of Data

#### 2.3.1. Data Registered by the Kinect Device

Two Kinect devices were used to record the subjects’ movement data. Each of these devices recorded the data of the complete skeletons of all participants in the session of 30 min. In order to register the information of the session for its later analysis, the application provided by Microsoft, Kinect Studio v2.0, was used. This application allows you to save all the information captured by Kinect, such as depth data, skeletons, sound, etc. for further analysis.

The computer application *ADHD Movements* was used for the analysis of the movement of all the subjects. The application estimates the distance covered by each joint of the skeleton during each minute of the session for each of the subjects.

**Figure 6:**
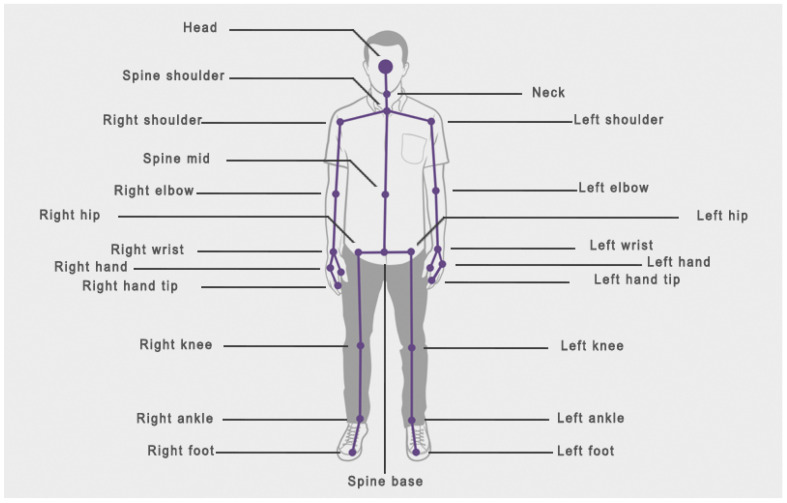
Joint map detected by Kinect.

As it has been previously mentioned, the Kinect device can detect up to 25 joints of 6 different bodies. Figure 6 shows the different joints detected by Kinect and their names.

The Kinect device detects 4 joints: *wrist*, *hand*, *hand tip* and *thumb* from each hand. Kinect uses these joints for the recognition of gestures in a specific situation: that a subject is standing in front of the Kinect, showing the hand to the device.

In the case of the study that has been carried out, with the subjects sitting in a natural position, that is, without clearly showing the hand to the device, it has been detected that the joint that is best recognized and registered of the four abovementioned, is the *wrist*. For this reason, in the results of this study, the *hand*, *hand tip* and *thumb finger* joints have been rejected.

Likewise, for the lower limbs, Kinect detects the *ankle* and *foot* joints. However, as shown by the research carried out by [23], this device detects skeletons in a standing position much better than in a sitting one. For this reason, after detecting that the recognition and registration of the *ankle* is much better than that of the *foot*, the *foot* has been rejected.

Thus, of the 25 joints registered by the Kinect device, only 17 have been analysed for the results of this study.

#### 2.3.2. Data Registered by Observers

Once all the editions of the study techniques workshops were made, a second observer visualized the data recorded by the Kinect and the times that the subjects leave seat and squirm in seat were registered in a template previously prepared.

These data were compared with those previously collected by the observer who attended the different sessions of the workshops. For the study of the concordance of the data of the two observers, the *Kappa* index was used [24].

#### 2.3.3. Statistical Analysis

In order to analyse the movement registered by observers between the experimental group and the control group, as well as to analyse the difference in movement registered by observers between the experimental group with and without taking their prescribed medication, the Student’s *t*-test was applied and *d* index proposed by Cohen [24] was included to measure the effect size of the differences found, since the *t*-test can erroneously detect statistically significant differences. The interpretation of this index is as follows: small effect size (0.20 ≤ *d* ≤ 0.50), moderate (0.51 ≤ *d* ≤ 0.80) and large effect size (*d* > 0.80).

In order to analyse the differences between averages of movement of the different joints recorded by Kinect within the experimental group with and without their prescribed medication, receiver operating characteristic curves (*ROC* curves) have been used in addition to the *t*-test.

The way of interpreting the area under the *ROC* curve is that a test with an area greater than 0.9 has higher accuracy, while between 0.7 and 0.9 it indicates a moderate accuracy, between 0.5 and 0.7, lower accuracy and 0.5 a chance result (Conzelmann et al. [21]).

Statistical packages *SPSS 24* and *MedCalc 12* were used for the statistical analysis.

## 3. Results

### 3.1. Average Differences in Movement for Every Joint in the Experimental Group and the Control Group

**Figure 7:**
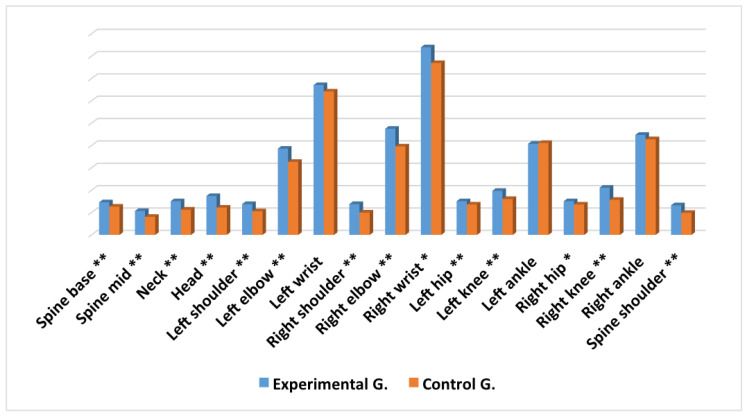
Average differences in movement for every joint in the experimental group and the control group. ** = *p* < 0.001; * = *p* < 0.05.

Results show that, except in the *left ankle* joint, the subjects with a firm diagnosis of ADHD obtained a higher average in the movement of the joints analysed, than those in the control group. The differences found between the two groups are significant for 14 of the 17 joints (Figure 7). The magnitude of the differences found was small in the joints: *spine mid*, *neck*, *head*, *left shoulder*, *left elbow*, *left wrist*, *right shoulder*, *right elbow*, *right knee* and *spine shoulder*, oscillating between 0.21 and 0.46 (Table 1). In the joints: *spine base*, *right wrist*, *left hip*, *left knee* and *right hip*, the differences found, although statistically significant, did not obtain the minimum levels required by [20] to be considered relevant.

### 3.2. Average Differences in Movement for Every Joint within the Experimental Group with and without Medication Intake

**Figure 8:**
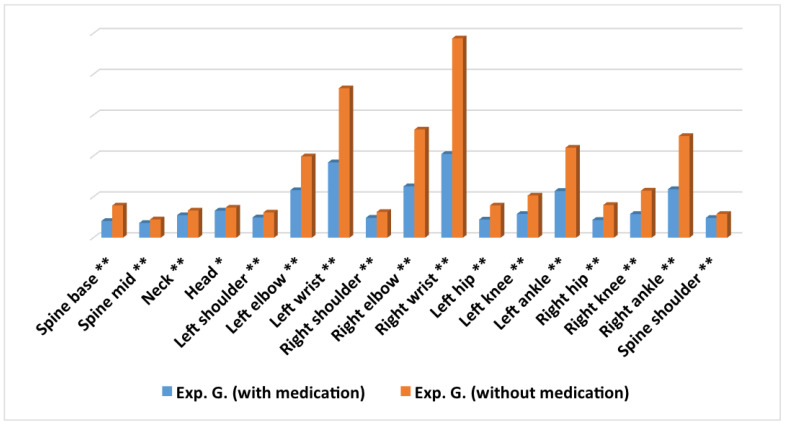
Average differences in movement for every joint within the experimental group with and without medication intake. ** = *p* < 0.001; * = *p* < 0.05.

The results revealed that there were statistically significant differences for the 17 joints analysed, being in all of them the movement average higher in the group without medication (Figure 8). The magnitude for the differences was small for the joints: *spine mid*, *neck*, *left shoulder*, *right shoulder* and *spine shoulder*, oscillating between 0.22 and 0.42 (Table 2). The magnitude for the differences was moderate for the joints: *left elbow*, *left hip*, *left knee*, *left ankle*, *right knee* and *right ankle*, oscillating between 0.56 and 0.79.

The magnitude of the differences was high for the joints: *spine base*, *left wrist*, *right elbow*, *right wrist*, and *right hip*, oscillating between 0.83 and 1.12.

In the *head* joint, although the difference found between both groups was statistically significant, it did not obtain the minimum levels required by Cohen to be considered relevant [25].

**Figure 9:**
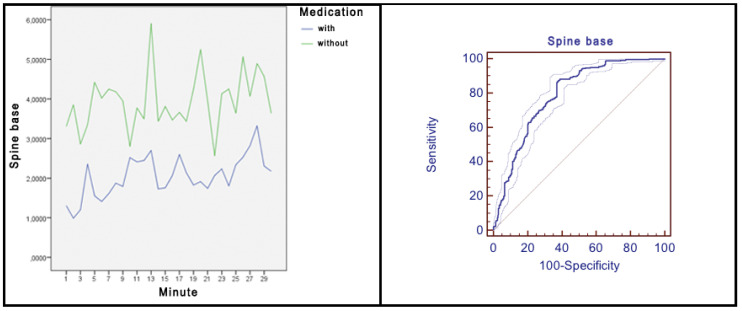
Spine base: movements average/minute and *ROC* curve.

In the joints *spine base* (Figure 9), *left elbow*, *left wrist*, *right elbow*, *right wrist*, *left hip*, *right hip* and *right ankle*, as shown in respective graphs, the differences in movement were constant in time and maintained over time. In addition, in most of the joints, there were large peaks in which, obviously, the amount of movement of the subjects without medication intake was much higher.

All of the above implies an area under the *ROC* curve with values of 0.79 for the *base spine*, 0.73 for the *left elbow*, 0.81 for the *left wrist*, 0.77 for the *right elbow*, 0.83 for the *right wrist*, 0.77 for the *left hip*, 0.79 for the *right hip* and 0.72 for the *right ankle*. For the rest of the joints analysed, except for *head*, *neck* and *spine shoulder* joints, the differences in movement were remarkable in practically all the minutes, but they did not remain constant over time. For the joints *head*, *neck* and *spine shoulder*, although there were peaks in which the amount of movement of the subjects without medication was much higher, at different times the group of subjects with medication recorded more movements than the group without taking medication.

### 3.3. Average Differences in Movement for Every Joint in the Experimental Group, According to Sex

**Figure 10:**
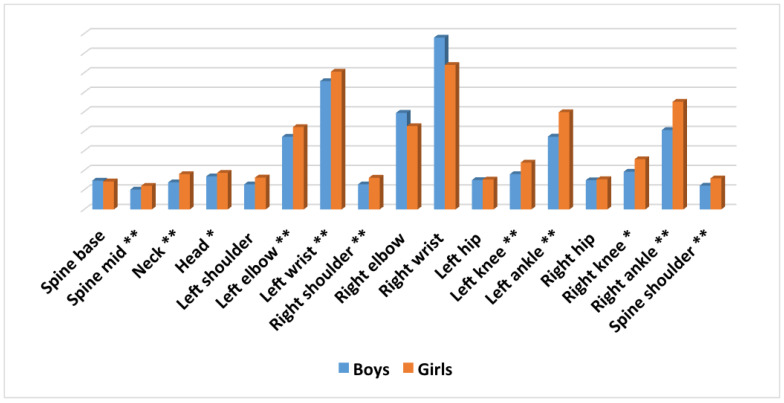
Average differences in movement for every joint in the experimental group, according to sex. ** = *p* < 0.001; * = *p* < 0.05.

Results showed that girls obtained a higher average in movement in comparison with boys for 14 of the 17 joints analysed (Figure 10). Thus, the only joints in which boys obtained a higher average are *spine base*, *right elbow* and *right wrist*.

The differences found between the two groups were significant for 11 of the 17 joints (Table 3).

### 3.4. Movement Difference Registered by Observers

This section offers an analysis of the difference in movement registered by observers for the movements *left seat* and *squirmed in their seat*. To evaluate the agreement between observers, Cohen’s *Kappa* index was calculated [24], obtaining a result of κ = 0.93. The results showed that for the two movements registered in the experimental group and in the control group (Table 4), the highest average corresponded to the experimental group, although only in the *squirm in seat* movement the average difference was significant. In this case, the magnitude of the difference found was moderate: *d* = 0.69.

On the other hand, results revealed that for the two movements registered in the experimental group with and without the medication intake, the highest average corresponded to the group without prescribed medication (Table 5), although only in the movement *squirm in seat* the difference of averages was significant, with a high magnitude: *d* = 0.80.

On the other hand, results revealed that for the two movements registered in the experimental group with and without the medication intake, the highest average corresponded to the group without prescribed medication (Table 5), although only in the movement *squirm in seat* the difference of averages was significant, with a high magnitude: *d* = 0.80.

## 4. Discussion

The identification of movement, posture, or a gesture made by a human body in real time is a difficult challenge because it has been found that the human body can perform a great deal of movement and it can have different size etc. [26]. Notwithstanding the above, after having carried out a series of tests with different technologies, the analysis of various technological devices, and the confirmation that Kinect has been previously used successfully in a wide range of research fields [27,28,29,30,31,32,33,34,35,36,37], it has been concluded that the Kinect V.2 is a suitable device to recognize and capture the movement of subjects in a teaching/learning situation, and thus achieve the objectives of this research.

Therefore, after having analysed existing technology, the computer application *ADHD Movements* was developed to detect the number of visible skeletons and, from each of them, analyse and draw on the screen each of the joints. From each joint that makes up all the skeletons, the application estimates the distance it covers in the 3D space during each minute. So, for each joint, the distance between the different 3D points of the trajectory of the joint in the movement has been calculated for each minute of the session.

Once the computer application was developed and verified, the movement of the subjects was captured.

Results show that there were significant differences in the amount of objective movement between a clinical group of subjects with ADHD and a control group, obtaining a higher average of movement in the experimental group in all the analysed joints (except in the *left ankle* joint). In addition, the differences found between the two groups are statistically significant in 14 of the 17 joints analysed.

In the *left ankle* joint, the control group obtained a higher movement average than the experimental group, although there was no statistically significant difference. This may be due to the dominance or preference for the use of the right or left leg of the subjects that have been part of the research groups.

From the revision of the literature it is concluded that this is the first study that counts the amount of movement in different joints with Kinect in order to find differences between a group of subjects with a firm diagnosis of ADHD and a control group. However, given the importance of hyperactivity in ADHD [7,38], objective measures of movement have been studied for more than a decade, although motion capture has almost always been done with accelerometers (actigraphy and inertial measurement units), with infrared systems placing some device in the body of the subjects or analysing only a part of the body [6,14,15,16,17,18,19].

In all the studies reviewed, children with ADHD showed more body movement than those without ADHD [6,14,39,40] so the results of the study coincide. As for the objective movement difference estimated in the group of subjects with ADHD with and without medication intake, the results show that there are statistically significant differences for the 17 joints analysed, being in all of them the average of movement higher in the group without taking medication. The results we have obtained are similar to those of previous studies that indicate that medicated ADHD children show significantly less motor activity than non-medicated ADHD children [41,42,43].

Along the lines of a recent study in which the effect of methylphenidate was studied in children with ADHD [44], and in which head movements were not found to be significantly different between the groups (medicated ADHD, non-medicated ADHD and control children), in the study, in the *head* joint, although the difference found between both groups is statistically significant, it does not obtain the minimum levels required by Cohen to be considered relevant [25].

The non-specificity of some diagnostic criteria defined by the DSM-V and the WHO for ADHD, such as *squirm in their seat*, means that although applications have been developed specifically to estimate the quantity of movement [45], the estimation of this depends on what the observer associates with the gesture. For this reason, both the movement to *leave the seat*, as well as the one previously mentioned, in the study, were registered by different observers.

### Limitations, Strengths, and Future Directions

It is necessary to mention that this work is not exempt from limitations that must be considered when interpreting the results and their implications, as well as in the preparation of future studies in order to solve them and increase the understanding of the findings.

Firstly, the number of participants in the experimental group (32) may restrict the ability to detect significant differences between the groups. This limitation must be taken into account nevertheless minimized if we consider that other studies have presented a lower sample size [46,47]. In addition, the experimental group consisted of 24 boys and 8 girls, that is, 75% of the sample of participants with a firm diagnosis of ADHD were male. This data is in accordance with current epidemiological studies that indicate that the prevalence rate is greater in male [48]. However, it limits the extrapolation of the results analysed according to the sex of the participants. For this reason, in order to check whether the results are scalable, it would be interesting for future research to use large samples of subjects.

Besides, related to the sample size is the fact that experimental group consisted of 24 subjects with a diagnosis of ADHD with a combined presentation and eight with a predominantly inattentive presentation, according to the DSM-V criteria. In this study we have only analysed hyperactivity as a core feature of ADHD [7,8], together with the conditions of compliance with the diagnostic criteria in order to classify a predominantly inattentive presentation in ADHD that excludes those related to hyperactivity. Therefore, it would be interesting that in the future the objective movement tests carried out in this study could be carried out also in ADHD subjects with a predominant hyperactive/impulsive presentation.

Secondly, in this study the Kinect V.2 sensor was used to record the movement of subjects due to its simplicity and effectiveness [20]. In addition, different studies carried out with the Kinect device in several fields show that, although this is not as accurate as some traditional measurement technologies in research laboratories, it provides a good quality relationship for motion tracking systems with respect to the length of the body segments, joint angles and the displacement of the joints in the different body gestures [23]. All the above is not an obstacle to emphasize that the device has a series of limitations that must be considered. Thus, the Kinect device shows excellent results in wide movements such as sitting or standing up, but it shows poor precision in fine or small movements such as closing a hand [49]. This fact has led us to dismiss the results of the *hand*, *hand tip*, *thumb* and *foot* joints. On the other hand, the Kinect device detects and records the human body better in standing position and in the study the data has been captured in real education/learning situations, where the subjects are sitting. For this reason, it is possible that in certain positions of the subjects (for example, crossing the legs) occlusions have occurred. In these cases, the skeleton detection software of the device interpolates the positions of the undetected joints, being able to produce slight deviations between the real position of a joint and the interpolated position.

## 5. Conclusions

The results indicate that for the two registered movements, the highest average corresponds to the experimental group with respect to the control group, although only in the movement *squirm in seat* the difference in averages is statistically significant.

In the same way, when comparing the movements registered by the observers between the experimental group with and without the medication, the highest average corresponds to the group without medication. Only in the movement *squirm in seat* the difference of averages is significant, with a high magnitude (*d* = 0.80).

The absence of significant differences in the movement *leave their seat* when comparing the experimental group with the control group, may be due to both the “novelty” of the teaching/learning situation (it was the first time they were in that classroom and with those speakers) or to the effect of the medication. However, the results of the comparison of the group with and without medication cannot be due to this reason, since the assistants already knew the speakers and the classroom of the study techniques workshop.

In any case, in the absence of previous studies in which a register of this type of movements associated with the diagnostic criteria of DSM-V and WHO for ADHD in a teaching/learning situation, we cannot compare results.

The above results lead to a series of conclusions:

1. The software developed (*ADHD Movements*) for the Microsoft Kinect V.2 device is valid to capture the movement of 17 joints of up to 6 subjects in a teaching/learning situation.
2. Students with ADHD present more movement and *squirm more in their seat*, than students without ADHD.
3. Students with a firm diagnosis of ADHD without the prescribed medication present more movement and *squirm more in their seat* than ADHD students with the prescribed medication.
4. ADHD students with and without taking their prescribed medication present a similar amount of movement in the *head* joint.
5. Girls with ADHD present more movement than boys with ADHD.

## Figures and Tables

**Table 1:** Average differences in movement for every joint in the experimental group and the control rgroup.

Kinect Joint	Levene Test		Experimental Group		Control Group		Statistical Significance and Magnitude Differences			
	F	*p*	M	DE	M	DE	t	g.l.	*p*	*d*
Spine base	45.42	<0.001	2.92	2.41	2.53	1.82	4.73	2512.24	<0.001	0.18
Spine mid	74.18	<0.001	2.14	1.76	1.62	1.05	9.59	2665.48	<0.001	0.34
Neck	64.04	<0.001	3.01	2.36	2.26	1.60	9.70	2612.67	<0.001	0.36
Head	102.41	<0.001	3.48	2.52	2.44	1.67	12.79	2628.94	<0.001	0.46
Left shoulder	46.76	<0.001	2.76	2.18	2.11	1.66	8.62	2496.40	<0.001	0.32
Left elbow	46.53	<0.001	7.72	6.06	6.54	4.79	5.50	2448.92	<0.001	0.21
Left wrist	53.83	<0.001	13.41	10.31	12.84	7.87	1.62	2497.42	0.105	-
Right shoulder	111.21	<0.001	2.76	2.25	2.00	1.33	10.97	2665.93	<0.001	0.39
Right elbow	15.46	<0.001	9.50	7.85	7.92	7.00	5.38	2265.50	<0.001	0.21
Right wrist	92.18	<0.001	16.79	14.08	15.38	10.05	3.00	2571.63	0.003	0.11
Left hip	27.87	<0.001	3.02	2.35	2.72	1.93	3.57	2393.97	<0.001	0.14
Left knee	4.35	0.037	3.95	4.00	3.21	4.49	4.26	1890.72	<0.001	0.18
Left ankle	10.13	0.001	8.16	7.72	8.23	8.70	−0.205	1883.48	0.838	-
Right hip	25.15	<0.001	3.02	2.34	2.72	1.97	3.47	2356.94	0.001	0.14
Right knee	24.75	<0.001	4.23	4.79	3.14	3.47	6.76	2554.91	<0.001	0.25
Right ankle	0.635	0.426	8.95	8.54	8.56	9.54	1.08	2666	0.279	-
Spine shoulder	73.26	<0.001	2.65	2.21	1.97	1.40	9.61	2650.59	<0.001	0.35

**Table 2:** Average differences in movement for every joint within the experimental group with and without medication intake.

Kinect Joint	Levene Test		Experimental Group (with Medication)		Control Group (with Medication)		Statistical Significance and Magnitude Differences			
	F	*p*	M	DE	M	DE	t	g.l.	*p*	*d*
Spine base	32.30	<0.001	2.05	1.83	3.94	2.60	16.92	1370.59	<0.001	0.83
Spine mid	0.001	0.974	1.80	1.57	2.53	1.89	8.61	1676	<0.001	0.42
Neck	<0.001	0.996	2.75	2.18	3.32	2.51	4.93	1676	<0.001	0.24
Head	4.63	0.031	3.31	2.45	3.68	2.59	3.02	1609.82	0.002	0.15
Left shoulder	0.381	0.537	2.48	2.04	3.08	2.29	5.69	1676	<0.001	0.28
Left elbow	26.33	<0.001	5.81	5.23	9.94	6.20	14.61	1526.61	<0.001	0.72
Left wrist	15.68	<0.001	9.21	8.60	18.28	9.99	19.75	1543.72	<0.001	0.97
Right shoulder	0.490	0.484	2.43	2.04	3.15	2.41	6.60	1676	<0.001	0.32
Right elbow	186.14	<0.001	6.28	4.87	13.23	8.91	19.39	1163.32	<0.001	0.95
Right wrist	147.05	<0.001	10.24	9.40	24.37	14.77	22.94	1281.24	<0.001	1.12
Left hip	13.56	<0.001	2.22	1.96	3.94	2.43	15.70	1492.09	<0.001	0.77
Left knee	102.38	<0.001	2.91	2.91	5.15	4.71	11.50	1256.51	<0.001	0.56
Left ankle	276.86	<0.001	5.71	4.61	11.01	9.44	14.25	1090.70	<0.001	0.70
Right hip	11.56	0.001	2.17	1.94	4.00	2.39	16.95	1494.15	<0.001	0.83
Right knee	212.30	<0.001	2.90	2.80	5.76	6.00	12.13	1064.31	<0.001	0.60
Right ankle	266.49	<0.001	5.93	5.37	12.43	10.07	16.12	1146.82	<0.001	0.79
Spine shoulder	0.074	0.786	2.42	1.99	2.91	2.41	4.49	1676	<0.001	0.22

**Table 3:** Average differences in movement for every joint in the experimental group, according to sex.

Kinect Joint	Boys		Girls		Statistical Significance and Magnitude Differences		
	M	DE	M	DE	F	*p*	*d*
Spine base	2.95	2.45	2.86	2.32	2.15	0.643	-
Spine mid	2.03	1.67	2.41	1.94	62.34	<0.001	0.22
Neck	2.77	2.15	3.62	2.72	152.60	<0.001	0.37
Head	3.38	2.42	3.74	2.73	127.52	<0.001	0.14
Left shoulder	2.56	2.02	3.26	2.47	1.14	0.286	-
Left elbow	7.44	6.03	8.43	6.06	27.58	<0.001	0.16
Left wrist	13.13	10.66	14.11	9.36	13.38	<0.001	0.10
Right shoulder	2.57	2.08	3.24	2.57	129.93	<0.001	0.30
Right elbow	9.89	8.40	8.53	6.15	2.26	0.132	-
Right wrist	17.59	15.29	14.79	10.19	3.45	0.063	-
Left hip	3.00	2.31	3.06	2.46	4.37	0.081	-
Left knee	3.61	3.67	4.80	4.64	41.07	<0.001	0.30
Left ankle	7.45	7.69	9.95	7.52	26.14	<0.001	0.33
Right hip	2.99	2.28	3.09	2.49	4.29	0.075	-
Right knee	3.86	4.54	5.14	5.25	5.50	0.019	0.30
Right ankle	8.12	7.98	11.02	9.50	14.16	<0.001	0.34
Spine shoulder	2.43	2.05	3.18	2.48	143.90	<0.001	0.34

**Table 4:** Average differences in movement registered by observers in experimental group and control group.

Movement	Levene Test		Experimental Group		Control Group		Statistical Significance and Magnitude Differences			
	F	*p*	M	DE	M	DE	t	g.l.	*p*	*d*
Squirm	0.82	0.367	8.50	4.71	5.27	4.65	2.77	63	0.007	0.69
Leave sit	0.25	0.614	1.35	3.59	1.12	3.38	0.268	62	0.790	-

**Table 5:** Average differences in movement for every joint within the experimental group with and without intake of prescribed medication.

Movement	Levene Test		Experimental Group (without Medication)		Control Group (without Medication)		Statistical Significance and Magnitude Differences			
	F	*p*	M	DE	M	DE	t	g.l.	*p*	*d*
Squirm	0.79	0.324	8.92	4.88	16.52	12.54	3.34	24	0.003	0.80
Leave sit	0.24	0.608	1.64	3.95	5.24	12.97	−1.62	24	0.118	-
